# A farewell to *R*: time-series models for tracking and forecasting epidemics

**DOI:** 10.1098/rsif.2021.0179

**Published:** 2021-09-29

**Authors:** Andrew Harvey, Paul Kattuman

**Affiliations:** ^1^ Faculty of Economics, University of Cambridge, Cambridge, UK; ^2^ Cambridge Judge Business School, University of Cambridge, Cambridge, UK

**Keywords:** COVID-19, Gompertz curve, Kalman filter, state-space model, stochastic trend, waves

## Abstract

The time-dependent reproduction number, *R*_*t*_, is a key metric used by epidemiologists to assess the current state of an outbreak of an infectious disease. This quantity is usually estimated using time-series observations on new infections combined with assumptions about the distribution of the serial interval of transmissions. Bayesian methods are often used with the new cases data smoothed using a simple, but to some extent arbitrary, moving average. This paper describes a new class of time-series models, estimated by classical statistical methods, for tracking and forecasting the growth rate of new cases and deaths. Very few assumptions are needed and those that are made can be tested. Estimates of *R*_*t*_, together with their standard deviations, are obtained as a by-product.

## 1. Introduction

The degree of infectiousness of a disease is given by the basic reproduction number, *R*_0_, defined as the number of infections that are expected to result from a single infectious individual in a completely susceptible population. As an infection spreads, immunity starts to develop and for serious diseases, such as coronavirus disease 2019 (COVID-19), social behaviour may change endogenously, or may be modified, perhaps by the imposition of lockdown and social distancing measures. The progress of an epidemic is then usually tracked by the effective, or instantaneous, reproduction number, *R*_*t*_, which is the number of people in a population who get infected by an individual at any specific time (e.g. [1–3]). Such tracking is of considerable importance for planning, but it raises the question of whether estimating *R*_*t*_ is to be regarded as an end in itself or as a means to an end, namely tracking and forecasting the number of new cases, hospital admissions and deaths.

Harvey & Kattuman [4]—hereafter HK—developed a class of generalized logistic (GL) time-series models for predicting future values of a variable which, when cumulated, is subject to an unknown saturation level. These models are relevant for many disciplines, but attention in HK was focused on applications for coronavirus.^1^ Observations on the cumulative series are transformed to growth rates and the logarithms of these growth rates are modelled with a time trend. Allowing this trend to be time varying introduces flexibility and enables the effects of changes in policy and the environment to be tracked by filters for the level and slope. The filters are functions of current and past observations implied by the model. They can produce nowcasts of the current level of the incidence curve, together with forecasts of its future direction. Estimation is by maximum likelihood (ML) and goodness of fit can be assessed by standard statistical test procedures.

The methods used by epidemiologists to assess the current state of an infectious disease use time-series observations on new infections, together with information on the distribution of the serial interval of transmissions, sometimes called the infection profile (e.g. [5–8]). A brief description of the method in [5] can be found in appendix A. Bayesian methods are often used to combine the information on the serial interval with the observations on new cases, often smoothed by a simple, but to some extent arbitrary, moving average. These formulae effectively link estimates of *R*_*t*_ to the growth rate in new cases, as do the more general formulae given in [1].

In our approach, estimates of the growth rate of new cases are produced directly by the time-series model from the raw data. The nowcasts and forecasts of *R*_*t*_, together with the equivalent of Bayesian credible intervals, therefore emerge as a by-product. The underlying assumptions are clear and are subject to diagnostic tests, so estimates of *R*_*t*_ are implicitly validated. In contrast to *R*_*t*_, which is not observed directly, the accuracy of forecasts of future observations can be assessed *ex post*, providing further testing of the effectiveness of the model.

The HK model is reviewed in §2 and in §3 it is shown how new cases growth rate estimates can be used to nowcast *R*_*t*_ and make short-term predictions. The implicit weights in the model-based filter are compared with the weights in the simple moving average ratio estimators used by the Robert Koch Institute, Germany (RKI). In §4, data from Germany and Florida are used to illustrate how the model is able to assess the importance of spikes in new cases and track second waves. Section 5 concludes by suggesting that tracking an epidemic by methods dependent on *R*_*t*_ may be neither necessary nor desirable: the focus should be on the growth rates of new cases and deaths, together with their predicted time path.

## 2. The dynamic Gompertz model and its implementation

The model in HK uses data on the time series of the cumulative total, *Y*_*t*_, of a target series, such as confirmed cases of a disease or deaths. HK show how the theory of GL growth curves suggests observational models of the form

2.1 $$\begin{array}{rl} & \ln y_{t}=\rho \ln Y_{t-1}+\delta +\gamma t+\varepsilon_{t},\\ & \;\rho \geq 1,\;\gamma <0,\;t=2,\ldots ,T, \end{array}$$

where *y*_*t*_ = Δ*Y*_*t*_ = *Y*_*t*_ − *Y*_*t*−1_ is the daily change and ɛ_*t*_ is a disturbance term. The model for the Gompertz curve is obtained by setting *ρ* = 1, but subtracting ln*Y*_*t*−1_ from both sides gives a simple time trend regression for the logarithm of the growth rate of the cumulated series, that is, ln*g*_*t*_ where *g*_*t*_ = *y*_*t*_/*Y*_*t*−1_ or Δln*Y*_*t*_.

**Remark 2.1.**

The growth curve for the ascending phase of an epidemic proposed in [9] implies an observational equation of the form (2.1) with *γ* = 0 and with *ρ* a deceleration parameter in the range 0 ≤ *ρ* ≤ 1; see also [7]. When *ρ* = 1 the cumulative total grows exponentially. The introduction of a time trend with *γ* < 0 gives sub-exponential growth.

Deterministic trends are too inflexible for most practical time-series modelling. A stochastic trend may be introduced into the equation for ln*g*_*t*_ and this extra flexibility allows *ρ* to be set to 1. The resulting dynamic Gompertz model is

2.2 $$\ln g_{t}=\delta_{t}+\varepsilon_{t},\;\varepsilon_{t}∼NID(0,\sigma_{\varepsilon }^{2}),\;t=2,\ldots ,\;T,$$

where^2^

2.3 $$\begin{array}{rl} & \delta_{t}=\delta_{t-1}+\gamma_{t-1}+\eta_{t},\;\eta_{t}∼NID(0,\sigma_{\eta }^{2}),\\ \mathrm{and}\; & \gamma_{t}=\gamma_{t-1}+\zeta_{t},\;\;\zeta_{t}∼NID(0,\sigma_{\zeta }^{2}), \end{array}\}$$

and the normally distributed and serially independent irregular, level and slope disturbances, ɛ_*t*_, *η*_*t*_ and *ζ*_*t*_ respectively, are mutually independent. When $\sigma_{\zeta }^{2}$ is positive but $\sigma_{\eta }^{2}=0$, the trend, *δ*_*t*_, is an integrated random walk (IRW). It is this form of the stochastic trend that turns out to be most useful for tracking an epidemic because it is the movements in slope *γ*_*t*_ which are crucial for that purpose. The key parameter is then the signal–noise ratio, $q=\sigma_{\zeta }^{2}/\sigma_{\varepsilon }^{2}$. A deterministic trend is obtained when *q* is zero. Other components, such as day of the week effects, may be included in the right-hand side of (2.2).

Stochastic trend models can be estimated using techniques based on state-space models and the Kalman filter (KF) [10,11]. Here the computations were performed using the STAMP package^3^ [12]. The KF outputs the estimates of the state vector (*δ*_*t*_, *γ*_*t*_)^′^. Estimates of the state at time *t* conditional on information up to and including time *t* are denoted (*δ*_*t*|*t*_, *γ*_*t*|*t*_)^′^ and given by the contemporaneous filter; the predictive filter, which outputs (*δ*_*t*+1|*t*_, *γ*_*t*+1|*t*_)^′^, estimates the state at time *t* + 1 from the same information set. It may sometimes be useful to review past movements by the smoother, denoted (*δ*_*t*|*T*_, *γ*_*t*|*T*_)^′^, which is the estimate of the state at time *t* based on all *T* observations. Estimation of the unknown variance parameters is by ML. Tests for normality and residual serial correlation are based on the standardized innovations, that is, one-step-ahead prediction errors, *v*_*t*_ = ln*g*_*t*_ − *δ*_*t*|*t*−1_, *t* = 3, …, *T*.

**Figure 1.**
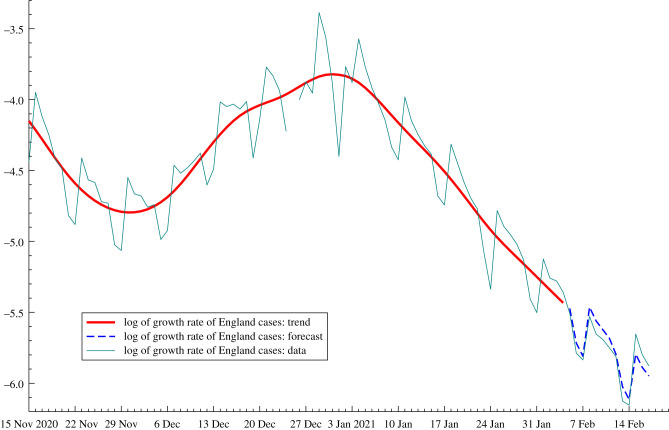
COVID-19 in England from early November 2020 until 17 February 2021. Trend from the fitted model is given by the bold line and the forecasts from 5 February 2021 are shown by dashes. The estimate of the trend and its slope on 4 February 2021 are *δ*_*T*|*T*_ = −5.433 and *γ*_*T*|*T*_ = −0.046, respectively.

Figure 1 shows data for the logarithm of the growth rate of the cumulated series of new cases of COVID-19 in England from early November 2020 until 17 February 2021.^4^ The model, which includes a day of the week component and a signal–noise ratio set to *q* = 0.005, was fitted to observations up to and including 4 February 2021. The bold line indicates the smoothed estimates of the trend and, as will be shown in the next section, it is the estimates of the trend and slope at the end of the series that provide the information needed to compute the nowcasts of *R*_*t*_. The dashed line shows the forecasts from 5 February 2021 onwards. As can be seen these two-week-ahead forecasts are successful in capturing the trend and day of the week movements. Recursions for making forecasts of the actual number of new cases, that is, the $y_{t}^{\prime }$s, are given in HK. However, the emphasis in this article is on nowcasting and forecasting of the growth rate of *y*_*t*_ and this only requires estimates of *δ*_*t*_ and *γ*_*t*_.

**Remark 2.2.**

When *y*_*t*_ is small, it may be better to specify its distribution, conditional on past values, as discrete. The usual choice is the negative binomial. The way in which a dynamic model may be constructed is set out in HK; software can be found in [13].

## 3. Tracking *R*

A simple and transparent estimator of *R*_*t*_ is

3.1 $${\hat{R}}_{k,\tau ,t}=\frac{\sum_{\;j=0}^{k-1}y_{t-j}}{\sum_{\;j=\tau }^{k+\tau -1}y_{t-j}}=\frac{\sum_{\;j=0}^{k-1}y_{t-j}}{\sum_{\;j=0}^{k-1}y_{t-\tau -j}}=\frac{\Delta_{k}Y_{t}}{\Delta_{k}Y_{t-\tau }},$$

where Δ_*k*_*Y*_*t*_ = *Y*_*t*_ − *Y*_*t*−*k*_, *k* = 1, 2, …. The lag of *τ* reflects the generation interval, which is the number of days that must elapse before an infected person can transmit the disease. The length of the moving average, *k*, determines the degree of smoothing; a value of *k* = 7 has the advantage of removing the day of the week effect but at the cost of a slower response. The rationale for ${\hat{R}}_{k,\tau ,t}$ comes from [5]. In Germany, the national figure used by RKI is based^5^ on setting *τ* = 4 and *k* = 4 or 7, but with some prior nowcasting of the data as described in [14].

A little algebraic manipulation shows

3.2 $${\hat{R}}_{k,\tau ,t}=1+\tau {\hat{g}}_{y,t}≃\exp(\tau {\hat{g}}_{y,t}),$$

where

$${\hat{g}}_{y,t}=\frac{1}{\tau }\frac{\Delta_{k}Y_{t}-\Delta_{k}Y_{t-\tau }}{\Delta_{k}Y_{t-\tau }}$$

is an implicit estimator of *g*_*y*,*t*_, the growth rate in *y*_*t*_, and the exponential approximation applies when ${\hat{g}}_{y,t}$ is small. In a dynamic Gompertz model, the growth rate of *g*_*t*_ is tracked by the filtered estimates of the slope, that is, *γ*_*t*|*t*_, while the growth rate itself is tracked by *g*_*t*|*t*_ = exp*δ*_*t*|*t*_. Following the continuous time argument^6^ leads to *g*_*y*,*t*_ being estimated as

3.3 $$g_{y,t|t}=g_{t|t}+\gamma_{t|t},\;t=t^{\prime },\ldots ,T,$$

where *t*′ is the time at which the estimates are deemed to be reasonably reliable. The nowcast of *R*_*t*_ suggested by equation (3.2) with *k* = *τ* is

3.4 $${\tilde{R}}_{\tau ,t}=1+\tau g_{y,t|t}\;\mathrm{or}\;{\tilde{R}}_{\tau ,t}^{e}=\exp(\tau g_{y,t|t}).$$

The RKI estimator for COVID-19 implies *τ* = 4.

A general formula linking *R*_*t*_ to *g*_*y*,*t*_ is given in [1]. When *g*_*y*,*t*_ is estimated by (3.3), the expression is

3.5 $${\tilde{R}}_{t}^{M}=\frac{1}{M(-g_{y,t|t})},$$

where *M*(.) is the moment-generating function of the serial interval distribution, defined as the time between the onset of symptoms in a primary case and the onset of symptoms in secondary cases. When the distribution is degenerate, so that all secondary infections occur after exactly *τ* days, ${\tilde{R}}_{t}^{M}=$ $\exp(\tau g_{y,t|t})$, which is the same as ${\tilde{R}}_{\tau ,t}^{e}$. When the serial interval has a gamma distribution with parameters *a* and *b*, implying a mean of *ab* and a variance of *ab*^2^, ${\tilde{R}}_{t}^{M}={\left(1+bg_{y,t|t}\right)}^{a}$. Keeping the mean constant and letting *b* → 0 confirms that ${\tilde{R}}_{t}^{M}=$ $\exp(\tau g_{y,t|t})$, where *τ* is the mean generation interval. Setting *a* = *b* = 2, which is consistent with some of the estimates of the mean and variance obtained for COVID-19, yields

$${\tilde{R}}_{t}^{M}={\left(1+2g_{y,t|t}\right)}^{2}=1+4g_{y,t|t}+4g_{y,t|t}^{2},$$

so, when *g*_*y*,*t*|*t*_ is small, ${\tilde{R}}_{t}^{M}≃1+4g_{y,t|t}={\tilde{R}}_{4,t}$. Finally, we note the observation in [1, p. 602] that exp(*τg*_*y*,*t*|*t*_) is an upper bound for ${\tilde{R}}_{t}^{M}$ in equation (3.5). Overall it seems that, if a single formula is to be adopted for COVID-19, ${\tilde{R}}_{4,t}$ or ${\tilde{R}}_{4,t}^{e}$ is not a bad choice.

### 3.1. Sampling variability of nowcasts

When *q*, the signal–noise ratio in the Gaussian IRW model, is treated as known, the distribution of *γ*_*t*_, conditional on current and past observations, is normal with a mean *γ*_*t*|*t*_ and a variance $\sigma_{\gamma ,t|t}^{2}$ that are produced by the KF. The growth rate of the incidence curve, *g*_*y*,*t*_, depends on *g*_*t*_ as well as *γ*_*t*_ but, as argued below, its contribution to the variability of *g*_*y*,*t*_ is dominated by that of *γ*_*t*_. When the variability in *g*_*t*_ is ignored, the probability that *R*_*t*_ exceeds 1, that is, $\mathrm{Pr}(\gamma_{t}>-g_{t|t})$ where *g*_*t*|*t*_ is treated as fixed, can be obtained directly from the conditional distribution of *γ*_*t*_. This probability does not depend on which equation estimates *R*_*t*_ from *g*_*y*,*t*|*t*_ and it does not depend on *τ*.

When *R*_*τ*,*t*_ is defined as 1 + *τg*_*y*,*t*_, its distribution, again conditional on current and past observations, is normal with mean 1 + *τg*_*y*,*t*|*t*_ and standard deviation (SD) *τσ*_*γ*,*t*|*t*_. On the other hand, the conditional distribution of $R_{\tau ,t}^{e}$ is lognormal with mean

3.6 $$E_{t}(R_{\tau ,t}^{e})=\exp(\tau (g_{t|t}+\gamma_{t|t}+\left(\frac{\tau }{2}\right)\sigma_{\gamma ,t|t}^{2}))$$

and SD

3.7 $${\mathrm{SD}}_{t}(R_{\tau ,t}^{e})=E_{t}(R_{\tau ,t}^{e})\sqrt{\left(\exp\tau^{2}\sigma_{\gamma ,t|t}^{2}-1\right)}.$$

Note that $\exp\tau^{\;2}\sigma_{\gamma ,t|t}^{2}-1≃\tau^{2}\sigma_{\gamma ,t|t}^{2}$, so, when *E*_*t*_(*R*_*τ*,*t*_) is close to 1, ${\mathrm{SD}}_{t}(R_{\tau ,t}^{e})$ will be very close to the SD_*t*_(*R*_*τ*,*t*_).

Why is the variability in *g*_*t*|*t*_ ignored? From equation (2.2), *g*_*t*_ = exp*δ*_*t*_ and, because *δ*_*t*_ is normal, *g*_*t*_ is lognormal with mean $\mu_{g,t|t}=\exp(\delta_{t|t}+0.5\sigma_{\delta ,t|t}^{2})$ and variance $\mathrm{Var}(g_{t})=\mu_{g,t|t}^{2}$ $\left(\exp\sigma_{\delta ,t|t}^{2}-1\right)$, where $\sigma_{\delta ,t|t}^{2}$ is the variance of *δ*_*t*_. However, $\sigma_{\delta ,t|t}^{2}$ is typically small so *μ*_*g*,*t*|*t*_ ≃ exp*δ*_*t*|*t*_ = *g*_*t*|*t*_ and $\mathrm{Var}(g_{t})≃$ $\mu_{g,t|t}^{2}\sigma_{\delta ,t|t}^{2}≃g_{t|t}^{2}\sigma_{\delta ,t|t}^{2}$. Now

$$\mathrm{Var}(g_{y,t})=\mathrm{Var}(g_{t})+\mathrm{Var}(\gamma_{t})+2\mathrm{Cov}(g_{t},\gamma_{t}),$$

but, although $\sigma_{\delta ,t|t}^{2}$ is usually larger than $\sigma_{\gamma ,t|t}^{2}$, the former is multiplied by $g_{t|t}^{2}$ to get Var(*g*_*t*_), whereas $\mathrm{Var}(\gamma_{t})=\sigma_{\gamma ,t|t}^{2};$ note that $\sigma_{\delta ,t|t}^{2}$ itself does not depend on the value of *g*_*t*|*t*_. Although *g*_*t*|*t*_ can be high near the beginning of an epidemic, it tends to fall quite rapidly and once the epidemic is underway it rarely exceeds 0.05. The example of Florida, where the second wave increases *g*_*t*|*t*_, shows that, even in this case, Var(*g*_*t*_) remains negligible compared with Var(*γ*_*t*_).

### 3.2. Predictions of *R*

Predictions of *R*_*t*_ in the dynamic Gompertz model can be made from predictions of *g*_*y*,*t*_, that is,

3.8 $$\begin{array}{rl} g_{y,T+\ell |T} & =\exp\delta_{T+\ell |T}+\gamma_{T+\ell |T}\\ & =\exp(\delta_{T|T}+\gamma_{T|T}\ell )+\gamma_{T|T},\\ \ell & =1,2,\ldots , \end{array}$$

where ℓ is the number of steps ahead. When *g*_*y*,*T*|*T*_ is positive, so any estimate of *R*_*T*_ given by (3.5) is greater than 1, there is still a saturation level for *Y*_*t*_ so long as *γ*_*T*|*T*_ is negative; for example as *T* → ∞, ${\tilde{R}}_{\tau ,T+\ell |T}^{e}\to \exp(\tau \gamma_{T|T})$. When *γ*_*T*|*T*_ is zero, the growth of *y*_*t*_ is exponential and in this case it is helpful to characterize it by the doubling time, ln2/*g*_*y*,*T*|*T*_ = 0.693 exp( − *δ*_*T*|*T*_). When *γ*_*T*|*T*_ is positive, as can happen at the start of a new wave, predictions of *g*_*y*,*t*_ should not be made from (3.8). However, it may still be useful to quote the doubling time based on *g*_*y*,*T*|*T*_.

If, as in the previous sub-section, it can be assumed that *g*_*t*_ is relatively small, the predictive distribution of *g*_*y*,*T*+ℓ_, and hence of *R*_*T*+ℓ_, is available because the conditional distribution of *γ*_*T*+ℓ_ given observations up to and including time *T* is Gaussian with mean *γ*_*T*+ℓ|*T*_ = *γ*_*T*|*T*_ and variance $\sigma_{T+\ell |T}^{2}$, as produced by the predictive equations of the KF.

**Remark 3.1.**

The ability to make predictions offers insight into how to deal with reporting delay, as described in [15, pp. 3–4]. If the observation at time *t* actually relates to an event ℓ days earlier, the current *R*_*t*_ is better estimated by an ℓ-step-ahead forecast. When *γ*_*T*|*T*_ is negative, this forecast will be less than the nowcast.

### 3.3. Weights

The filtered estimates of *g*_*t*_ and *γ*_*t*_ in the dynamic Gompertz model, equation (2.2), are obtained by discounting past observations, with the rate of discounting depending on the signal–noise ratio, *q*. Weights implied by the KF and smoother for estimated states in a linear model can be obtained as output from the STAMP package, using a method described in [16]. The forcing variable in the filter is ln*g*_*t*_ and the weights assigned to it in the contemporaneous filter are the weights for *γ*_*t*|*t*_ plus the weights for *g*_*t*|*t*_. When *Y*_*t*_ is much larger than *y*_*t*_, as will be the case when an epidemic has been underway for some time, *g*_*t*|*t*_ will be relatively small and attention can be focused on *γ*_*t*|*t*_. Then, if the weights for the slope, *γ*_*t*|*t*_, are denoted *w*_*j*_, *j* = 0, 1, 2, …,

3.9 $$g_{y,t|t}≃\gamma_{t|t}=\sum_{\;j=0}^{t-2}w_{\;j}(\ln y_{t-j}-\ln Y_{t-j-1})≃\sum_{\;j=0}^{t-2}w_{\;j}\ln y_{t-j},$$

where the last approximation follows because ln*Y*_*t*−*j*−1_ is assumed to be changing very slowly and $\sum_{\;j=0}^{t-2}w_{\;j}=0$. When multiplied by *τ*, the weights in equation (3.9) feed directly into the estimators of *R*_*t*_ implied by equation (3.4). In particular

3.10 $${\hat{R}}_{\tau ,t}^{e}=\prod_{\;j=0}^{t-2}y_{t-j}^{\tau w_{\;j}}=\frac{\prod_{w_{\;j}\geq 0}y_{t-j}^{\tau w_{\;j}}}{\prod_{w_{\;j}<0}y_{t-j}^{\tau w_{\;j}}},$$

which is similar in form to (3.1) but with summations replaced by products.

**Figure 2.**
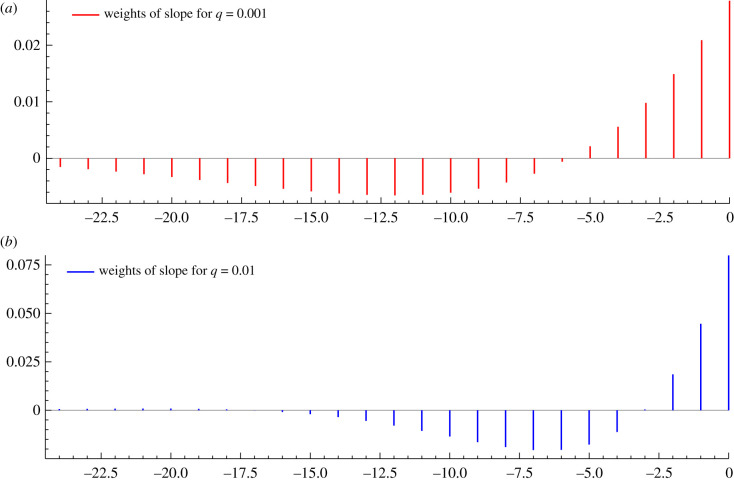
Weights assigned to ln*g*_*t*_ by the filter for the slope with (*a*) *q* = 0.001 and (*b*) *q* = 0.01.

Figure 2 shows the weights for the slope produced when *q* is 0.001 and 0.01. ML estimates of *q* are typically between these values. Setting *q* = 0.005, which has the first four weights positive and the next 17 negative, is a reasonable default. A higher value gives a faster response, which may be appropriate when there is a sharp change in the environment, perhaps because of a change in policy. However, it comes at the cost of making nowcasts and forecasts less stable.

### 3.4. The early phase of an epidemic

Any modelling is very difficult at the start of an epidemic because of the lack of data; see, for example, the remarks in appendix 3 of [5]. In the dynamic trend model initializing the KF in the absence of prior information is generally done with a non-informative (diffuse) prior on the level and slope, as in the STAMP package; alternatively they can be estimated as unknown parameters. However, in the early part of an epidemic the growth is exponential or very close to it; see, for example, the analysis of the 1918 outbreak of Spanish flu in [17]. Thus, we could set *γ*_0_ = 0. The filter for *δ*_*t*_, the level of ln*g*_*t*_, can have a prior distribution informed by knowledge about the basic reproduction number, *R*_0_. A rough estimate of ln*g*_0_ is then given from ${\hat{g}}_{0}=(1/\hat{\tau })\ln{\hat{R}}_{0}$ or ${\hat{g}}_{0}=({\hat{R}}_{0}-1)/\hat{\tau }$. Choosing a suitable variance for *δ*_0_ is more problematic.

## 4. Waves and spikes

After an epidemic has peaked, daily cases start to fall and the concern shifts to the possibility of a second wave and the need to deal with outbreaks indicated by spikes in the data so that they do not morph into waves. The monitoring of waves and spikes raises different issues, primarily because a wave applies to a whole nation or a relatively large geographical unit, whereas a spike is localized.

### 4.1. Spikes

**Figure 3.**
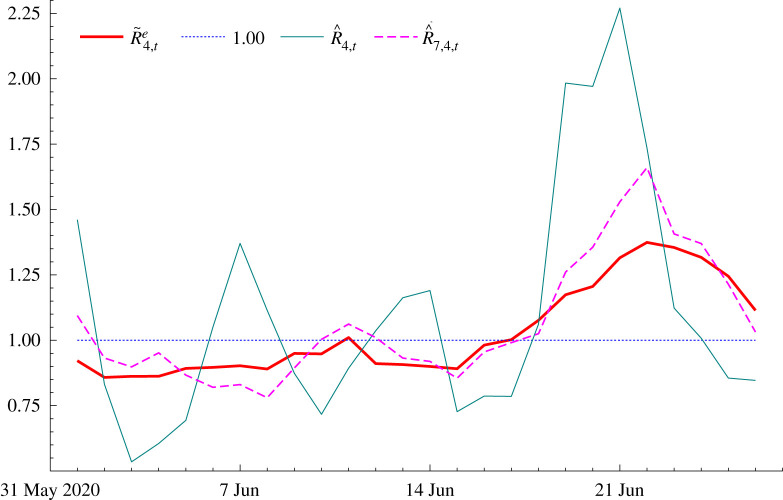
*R*_*t*_ for German new cases in June 2020.

When national numbers are low, a localized outbreak can also result in a jump in the national estimate of *R*_*t*_. However, such a jump does not indicate that there has been a sudden change in the way the infection spreads and so has few implications for overall policy. Figures for new cases in Germany show a sharp increase towards the end of June 2020, caused by an outbreak at a meat-processing factory in the Gütersloh area in Westphalia. Estimates produced by RKI at the time showed a big increase in *R*_*t*_, accompanied by what seems to us to be a rather narrow credible interval. Figure 3 compares the model-based reproduction number estimate, ${\tilde{R}}_{4,t}^{e}$, with the 4-day and overlapping 7-day moving average estimates, ${\hat{R}}_{4,t}$ and ${\hat{R}}_{7,4,t}$. The ${\hat{R}}_{4,t}$ estimates are very erratic and seriously affected by the failure to take account of the daily pattern. Estimates for Sundays and Mondays are typically lower. The peak in ${\hat{R}}_{4,t}$ has observations for Wednesday to Saturday in the numerator. Although ${\hat{R}}_{7,4,t}$ irons out some of the daily movement, the estimate of *R*_*t*_ is still affected. The model-based ${\tilde{R}}_{4,t}^{e}$ evolves more smoothly. After June the data give no indication of a sustained increase in new cases so the jump in estimates of *R*_*t*_, particularly ${\hat{R}}_{4,t}$, can safely be classed as a spike.

The model was estimated using data from 25 March to 26 June 2020, using cases data sourced from the European Centre for Disease Prevention and Control (ECDC) website.^7^ Estimates obtained using RKI’s nowcast data are not very different.^8^ The fit was good with very little evidence of residual serial correlation; the *Q*(15) statistic is 9.58. A Gaussian distribution seems a good approximation because the Bowman–Shenton test statistic, which is asymptotically distributed as $\chi_{2}^{2}$ under the null hypothesis, is only 0.77. The estimate of *q* was 0.0026.

The SD of the conditional distribution of *γ*_*t*_ is 0.0276. Thus the SD of *R*_*t*_ = 1 + 4*γ*_*t*_ is 0.110. For $R_{4,t}^{e}$ setting $E_{t}(R_{4,t}^{e})=1$ gives ${\mathrm{SD}}_{t}(R_{4,t}^{e})=0.111$, so the probability that it lies in the interval^9^ [0.895, 1.117] is 0.68. It makes little difference whether *R*_*t*_ is taken to be normal or lognormal. As regards the contribution of *g*_*t*_ to the variability *g*_*y*,*t*_, the 26 June value of *g*_*T*|*T*_ was only 0.0030 and SD(*g*_*T*_) was less than 1% of the SD of *γ*_*T*_.

### 4.2. Waves

The US state of Florida, the third most populous in the USA with a population of around 20 million, provides an example of a second wave. A graph of daily new cases^10^ from early March until 19 July 2020 shows a peak in early April followed by a steady decline. This is similar to the pattern for Germany and reflects the fact that Florida, like Germany, was in lockdown during April 2020. After April restrictions in Florida were eased there was a levelling out in May 2020, followed by a sharp rise in June.

**Figure 4.**
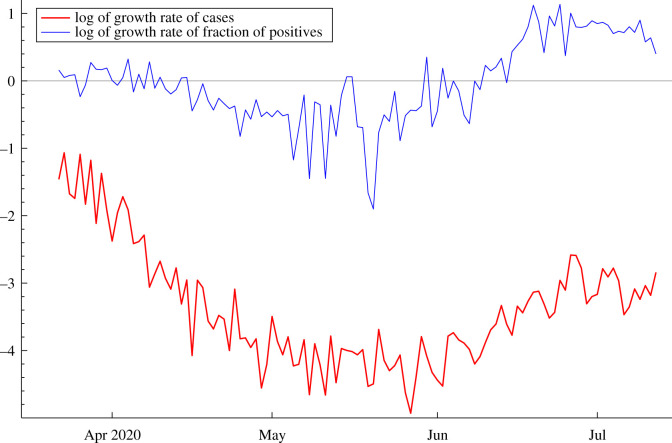
Logarithm of the growth rate of the total number of confirmed cases in Florida, together with the logarithm of the growth rate of the fraction of positives out of the total tested.

Figure 4 shows the logarithm of the growth rate of the number of confirmed cases, deaths and fraction of positives, starting 22 March 2020. (Before 22 March the data are very erratic.) After May there was an increase in testing. However, the growth rate in tests is roughly constant from the end of May onwards and this shows up in figure 4, where the logarithm of the growth in the proportion of positives follows a similar path to that of the logarithm of the growth in total cases. This suggests that confirmed cases are still a good indicator of the path of new infections.

**Figure 5.**
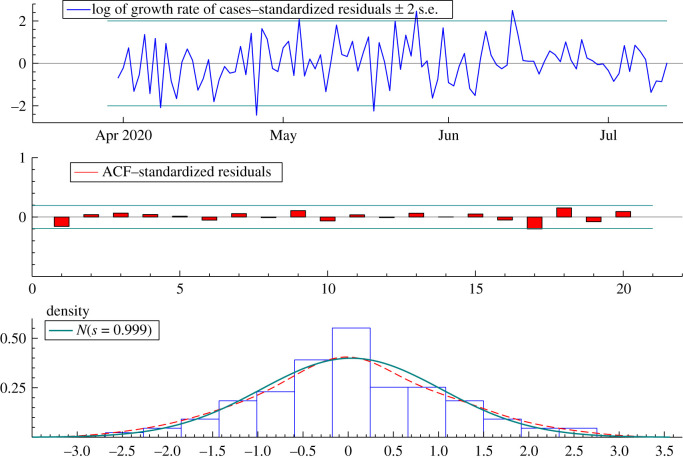
Residuals from fitting the model to the logarithm of the growth rate of Florida cases.

Fitting the dynamic Gompertz model, with a daily component, to data on confirmed cases from 22 March to 12 July 2020 gave residuals with very little residual serial correlation as the *Q*(16) statistic was only 8.42. The Bowman–Shenton test statistic was only 0.11 so a Gaussian distribution cannot be rejected. Graphical confirmation for the good fit is provided by figure 5.

**Figure 6.**
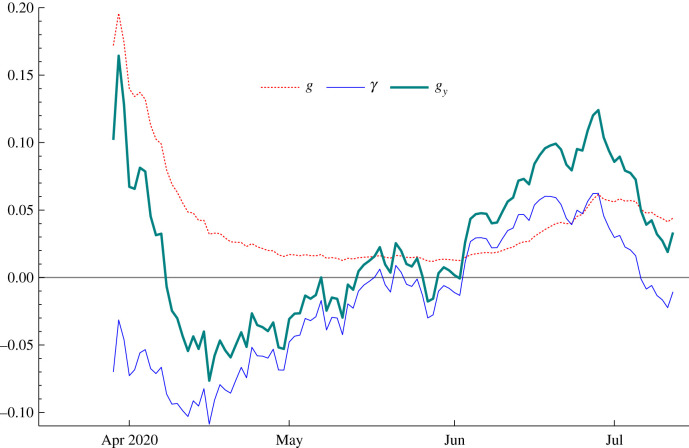
Filtered estimates of the growth rate, *g*_*t*_, and slope, *γ*_*t*_, for confirmed cases in Florida.

The signal–noise estimate, *q*, was 0.0014. Figure 6 shows the filtered estimates of *g*_*t*_ and *γ*_*t*_. At the beginning of June, *γ*_*t*|*t*_ becomes positive and its sharp rise is accompanied by an attendant rise in *g*_*t*|*t*_. The increase in *γ*_*t*|*t*_ continues until the end of June, when it changes direction and *g*_*t*|*t*_ peaks. The implied time series of nowcasts of *R*_*t*_ follows directly from their sum, *g*_*y*,*t*|*t*_. Thus ${\tilde{R}}_{4,t}^{e}$ reaches 1.5 by the end of June 2020 and then falls in July so that ultimately ${\tilde{R}}_{4,t}^{e}≃1.1$.

The filtered estimates of *g*_*t*_ and *γ*_*t*_ for confirmed cases in Florida are very different from those for Germany in that *g*_*t*|*t*_ no longer becomes negligible with time. Indeed for most of June it is of a similar order of magnitude to *γ*_*t*|*t*_. Nevertheless its contribution to the variability of *g*_*y*,*t*_ is still negligible. The SD of the conditional distribution of *γ*_*t*_ is 0.0275 while that of *δ*_*t*_ is 0.1296, translating into a SD of 0.0057 for *g*_*t*_. If the covariance term is ignored, the SD of *g*_*y*,*t*_ is 0.0281, which is only a little above the SD of *γ*_*t*_.

Re-estimating the model with another week of data has *γ*_*T*|*T*_ down to −0.031 but the SD is little changed at 0.027. The estimate of *g*_*T*_ is 0.034, giving ${\tilde{R}}_{4,T}^{e}=1.01$. Finally estimating using data up to 12 August leaves *γ*_*T*|*T*_ virtually unchanged but, because *g*_*T*|*T*_ is down to 0.011, ${\tilde{R}}_{4,T}^{e}$ is below 1, with a value of 0.91.

## 5. Conclusion

New time-series models are able to track the progress of an epidemic by providing nowcasts and forecasts of the daily number of new cases and deaths. Estimates and forecasts of the instantaneous reproduction number *R*_*t*_ can be computed as a by-product, using a formula that links it to the estimated growth rate of new cases, based on assumptions made about the serial interval distribution. The availability of the full conditional distribution allows the variability of the estimates to be assessed.

Current methods for tracking *R*_*t*_ do not pay due attention to the time-series properties of the data, whereas the method described in this paper is based on time-series techniques that have been shown to be effective in a range of disciplines. The dynamic response depends on a signal–noise ratio that can be estimated from the data rather than being inferred from knowledge about the serial interval of infections. An important element in time-series methodology is diagnostic checking and the fit of the model. We show how diagnostic methods can be applied in the context of epidemics and in doing so we raise questions about some of the assumptions, explicit or implicit, that are currently made in the estimation of *R*_*t*_. The ability of the model to track spikes and waves is illustrated with COVID-19 data from Germany and Florida.

We stress again that computing *R*_*t*_ is a by-product of our approach. Information on *R*_0_ could be used at the start of an epidemic, but with a dynamic time-series model its impact soon wears off. After that, calculations involving *R*_*t*_ play no part in nowcasting and forecasting daily cases and deaths.

## Endnotes

^1^
The models have been used as the basis for weekly trackers providing forecasts for regions and nations in the UK since the early part of 2021 (see https://www.niesr.ac.uk/latest-covid-19-tracker-0); and for states in India since May 2021 (see https://www.jbs.cam.ac.uk/covid-india). Past issues of both trackers are available on the websites.

^2^
HK had a negative sign in front of *γ* in (2.1) and (2.2) because in a growth curve the growth rate is always falling so it is more convenient to let *γ* be positive. This ceases to be the case once there are second waves.

^3^
A fully documented *R* script to carry out the estimation will be available shortly. An *R* script with the essential code is presented in the electronic supplementary material.

^4^
Sourced from: https://coronavirus.data.gov.uk/.

^5^
Erläuterung der Schätzung der zeitlich variierenden Reproduktionszahl R. Robert Koch Institute, 15 May 2020.

^6^
The continuous-time incidence curve is *μ*′(*t*) = *g*(*t*)*μ*(*t*), where *μ*(*t*) is the growth curve and *g*(*t*) is its growth rate. Taking logarithms and differentiating provides the rationale for our formula for *g*_*y*,*t*|*t*_; see the discussion surrounding eqn 6.1 in HK.

^7^
Sourced from: https://www.ecdc.europa.eu/en/publications-data/download-todays-data-geographic-distribution-covid-19-cases-worldwide.

^8^
Sourced from: https://www.rki.de/DE/Content/InfAZ/N/Neuartiges_Coronavirus/Projekte_RKI/Nowcasting.html.

^9^
Since *R*_*t*_, like *γ*_*t*_, is a random variable this is not, strictly speaking, a confidence interval. In a fully Bayesian framework, it would be called a credible interval.

^10^
Data on Florida are sourced from: https://covidtracking.com/data.

## Data accessibility

Data employed in this study are publicly available. The data for reproducing figure 1 in the paper were downloaded from https://coronavirus.data.gov.uk/. The data are provided as electronic supplementary material.

## Appendix A. Cori *et al.* [5] method for estimating *R*_*t*_

Following Cori *et al.* [5], Thompson *et al*. [6] generate an estimate of the current level of new cases, *y*_*t*_, that combines the estimate of *R*_*t*_ with an estimate, $\Lambda_{t|t-1}$, of the previous level based on the sum of new cases in the previous time period weighted by the infectivity function, or infectious profile after infection, *f*_*j*_, *j* = 0, 1, 2, …. This estimate can be written $\Lambda_{t|t-1}=\sum_{\;j=1}^{t}f_{\;j}y_{t-j}$, where $\sum_{\;j=\tau }^{t}f_{\;j}=1$ with *f*_*j*_ describing the serial distribution. This distribution is based on prior knowledge, such as data collected from household studies in the early phase of an infection. The estimate of *R*_*t*_ is obtained by Bayesian methods. Cori *et al.* [5, appendix 1] assume a Poisson distribution for *y*_*t*_ and a (conjugate) gamma prior for *R*_*t*−1_ with parameters *a* and *b*. The posterior mean of *R*_*t*_—its nowcast—is then

$${\hat{R}}_{k,t}^{*}=\frac{a+\sum_{\;j=0}^{k-1}y_{t-j}}{b^{-1}+\sum_{\;j=1}^{t-1}\Lambda_{t-j|t-j-1}}=\frac{a+\sum_{\;j=0}^{k-1}y_{t-j}}{b^{-1}+\sum_{\;j=1}^{t-1}\sum_{i=1}^{\;j}f_{\;j}y_{t-i}}=\frac{a+\sum_{\;j=0}^{k-1}y_{t-j}}{b^{-1}+\sum_{\;j=1}^{t}w_{\;j}y_{t-j}},$$

while the associated nowcast for the mean of new cases is

$${\hat{\mu }}_{t|t}={\hat{R}}_{k,t|t}^{*}\Lambda_{t|t-1}=\frac{a+\sum_{\;j=0}^{k-1}y_{t-j}}{b^{-1}+\sum_{\;j=1}^{t}w_{\;j}y_{t-j}}\sum_{\;j=1}^{t}f_{\;j}y_{t-j}.$$

It is proposed in [6] that *a* = 1 and *b* = 5 at the outset so that both the mean, *ab*, and SD, *ab*^2^, are set to 5. With no prior information *a* = 1/*b* = 0.

The numerator in ${\hat{R}}_{k,t}^{*}$ provides an estimate of the level at time *t* based on the last *k* observations. The value of *k* reflects a trade-off between response and stability. The choice of equal weights seems to be arbitrary. The weights in the second term reflect the structure implied by the sometimes imperfect knowledge of the distribution of the serial interval.

Cori *et al.* [5, appendix 2] suggest letting the summation in the denominator of equation (A 1) start at *j* = *τ*, where *τ* is the generation interval. Approximating the weights by a simple moving average of length *k* and assuming no prior information then gives equation (3.1), which is the formula for the instantaneous reproduction number used by RKI. The value of *k* may be set equal to the length of the serial interval.

In our time-series approach, the lag structure depends solely on the observations, *y*_*t*_, and the properties of the fitted model. The weights in figure 2 are comparable with those used to construct ${\hat{R}}_{k,t}^{*}$ in equation (A 1), except that they are multiplicative in the implied estimator of $R_{t}^{e}$. The negative weights in figure 2 correspond to the weights in the denominator of ${\hat{R}}_{k,t}^{*}$.
